# 

**Published:** 1863-04

**Authors:** 


### NOTICE.

At the last meeting of the National Dental Association each and every local Association represented in that body, or desiring to be represented, were requested to furnish me, as Secretary of the Association, with a statement of the condition of their respective Societies, embracing the following facts, viz: When the Society was formed; how often it meets; where the meetings are held; how many members in good standing there are, and their names; the names of the officers of each; how many members are members of the "National Association," and how many new delegates are appointed, and their names; and any other items that may be of interest.

I hope to receive these reports at the earliest possible period, that there may be time to make up the report for the National Association. J. TAFT, *Sec'y. of N. D. A*

### ASSOCIATION.

The Cincinnati Dental Association has had some very interesting meetings recently, the proceedings of which we have not had time to report. We should be pleased to welcome any of our professional brethren who can make it convenient to be with us at our meetings. There is now under way a work of some importance in the Society, in which we would be pleased to have the counsel of as many as possible of our brethren. Come in and help us. T.

### AGENT.

Mr. E. T. Lovering is authorized to act as agent for the Dental Register of the West, in the East and North-eastern States. Any arrangements made with him in reference to subscriptions or advertisements for the Register will be valid. J. TAFT,
*Publisher.*

### CONTENTS.

### DENTAL REGISTER,

### OF THE WEST.

Issued Monthly, at $3 a year in advance.

DEVOTED TO THE INTERESTS OF THE DENTAL PROFESSION.

**Edited by J. TAFT and GEO. WATT.**

The Volume commences in January and closes in December.

The Register being issued monthly is always fresh, therefore indispensable to the practitioner who desires to keep posted up with the new inventions and improvements which are daily developed. Neither expense nor effort will be spared to give value and variety to its contents. Articles will be illustrated with well executed engravings, whenever they will add to the interest or assist in explanation.

Its extensive circulation and frequency of issue makes it the BEST DENTAL ADVERTISING MEDIUM in the United States.

☞CONTRIBUTIONS SOLICITED.

Address Original Communications to **Geo. Watt, Xenia, O.**

Exchanges to **J. Taft**, or **Dental Register, Cincinnati, Ohio.**

Business Communications to

J. TAFT, Publisher,
172 Race Street, Cincinnati, O.

**JNO. T. TOLAND, Proprietor,**
38 West Fourth Street, Cincinnati, O.

Communications must reach the Editor as early as the 15th, to insure insertion in the next issue.

